# The Collateral Damage of the Pandemic on Non-COVID Related Pneumothorax Patients: A Retrospective Cohort Study

**DOI:** 10.3390/jcm11030795

**Published:** 2022-02-01

**Authors:** Wongi Woo, Bong Jun Kim, Ji Hoon Kim, Sungsoo Lee, Duk Hwan Moon

**Affiliations:** Department of Thoracic and Cardiovascular Surgery, Gangnam Severance Hospital, College of Medicine, Yonsei University, Seoul 06273, Korea; woopendo@gmail.com (W.W.); claris@yuhs.ac (B.J.K.); hoon01041@yuhs.ac (J.H.K.); chestlee@yuhs.ac (S.L.)

**Keywords:** pneumothorax, spontaneous pneumothorax, tension pneumothorax, COVID-19, SARS-CoV-2, collateral damage, pandemic

## Abstract

Background: Since the onset of the COVID-19 pandemic, there have been many reported cases showing the consequences—or the collateral damages—of COVID-19 on patients with non-COVID-related diseases. This study aimed to compare the clinical manifestations and treatment results of non-COVID-related pneumothorax patients before and during the pandemic. Methods: We retrospectively reviewed non-COVID-related pneumothorax patients who visited our hospital before the onset of the pandemic and during the pandemic. The primary outcome was the difference in the amount of pneumothorax between the two periods, and the secondary outcome was the difference in the treatment results between them. Multivariable logistic regression was conducted to find risk factors related to massive pneumothorax. Results: There were 122 and 88 patients in the pre-pandemic and pandemic groups, respectively. There was no significant difference between the two groups with respect to the preoperative demographic variables. However, the median amount of pneumothorax was significantly higher in the pandemic group (pre-pandemic: 34.75% [interquartile range (IQR) 18.30–62.95] vs. pandemic: 53.55% [IQR 33.58–88.80], *p* < 0.0001) and massive pneumothorax were more frequent in the pandemic group (52.3% vs. 30.3%, *p* = 0.002). Furthermore, more patients experienced re-expansion pulmonary edema after treatments during the pandemic (*p* = 0.0366). In multivariable analysis, the pandemic (OR: 2.70 [95% CI 1.49–4.90], *p* = 0.0011) was related to the occurrence of massive pneumothorax. Conclusion: During the pandemic, patients presented with a larger size of pneumothorax and had more re-expansion pulmonary edema, even in a country that handled the COVID-19 pandemic relatively well.

## 1. Background

The COVID-19 pandemic has severely impacted the health care system worldwide. In many countries, hospitals reached their capacity limits due to surges in COVID-19 cases, significantly impacting non-COVID-19 patients. It has been estimated that more than 28 million surgeries have been canceled worldwide [1]. Moreover, the overall visits to outpatient clinics have also distinctly decreased in Europe [2]; similarly, the Kaiser foundation in the United States has seen a 69% reduction of non-COVID hospital admissions during the first four months of the pandemic [3].

Non-COVID-related care has sharply decreased largely due to hospitals reaching their capacity limits and the social distancing policies set by governments. Additionally, and perhaps with greater influence, fear of COVID-19 infection among patients has also played a key role in the decrease of non-COVID-19-related hospital visits and admissions. These additional impacts of the pandemic, which ultimately will have detrimental effects on the long-term prognosis of patients, are referred to as the “collateral damage of COVID-19”, at least within the field of medicine [3]. The collateral impact of COVID-19 would have a great influence on patients with chronic medical conditions, especially cancer patients. Several studies have reported that delays in surgery among cancer patients impacted their long-term survival, and the proportion of advanced stages of malignancies increased during the pandemic [4]. On the other hand, as the pandemic continues over a long period, there were rising concerns for non-COVID-19 patients requiring urgent care, such as acute coronary syndrome, stroke, or appendicitis [5,6,7,8]. During the national lockdown in Italy, COVID-19 changed the quality of emergent surgical care with poorer prognosis and higher mortality rates [9]. In the field of thoracic surgery, the national survey among Spanish thoracic surgeons reported decreased surgical activity in oncologic and emergent surgery [10]. Although there were previous reports of pneumothorax after SARS-CoV-2 infection [11,12,13], there has not been a study of non-COVID pneumothorax patients who often require emergent treatment that could be affected by the hospital capacity during the pandemic.

In this study, based on anecdotal reports of a decrease in pneumothorax patients, we investigated the collateral impact of the pandemic on non-COVID-19 related pneumothorax patients. We hypothesized that patients with pneumothorax would present with an increased amount of pneumothorax during the pandemic due to the inability or hesitation to visit hospitals.

## 2. Patients and Methods

### 2.1. Patients Selection

From January 2019 to May 2021, we retrospectively reviewed 443 patients who visited our hospital in the Gangnam district, Seoul, Korea. The pre-pandemic period was defined from 1 January to 31 December 2019, and the pandemic period was defined from 1 March 2020 to 31 May 2021. All patients during the pandemic were negative on the polymerase chain reaction test for SARS-CoV-2. Patients with primary spontaneous and secondary pneumothorax were included. We excluded patients who visited our hospital from January to February 2020 because the level of restrictions imposed by the South Korean government was low at that time, and the pandemic was technically declared on 11 March 2020, by the World Health Organization.

**Figure 1:**
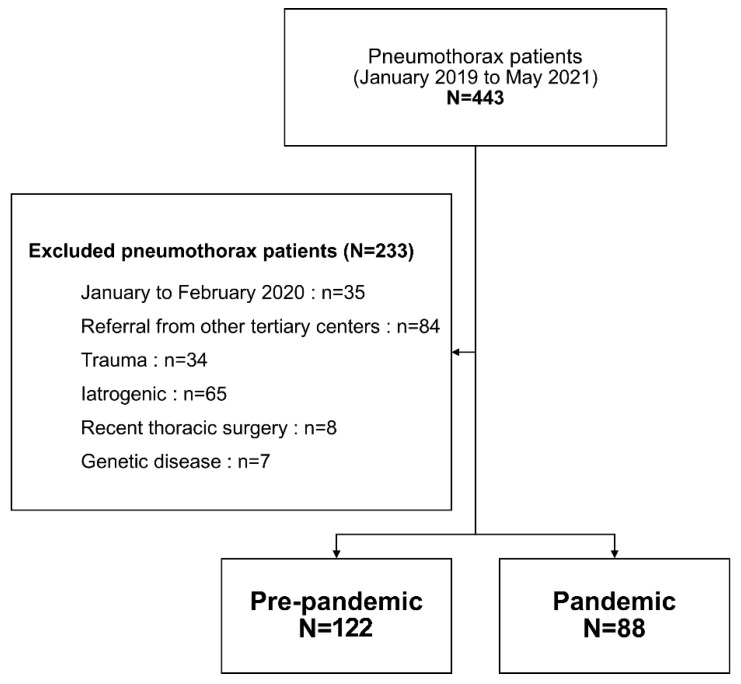
Study diagram describing patients’ inclusion process.

In addition, we excluded iatrogenic, traumatic, or catamenial pneumothorax and patients who were referred from other tertiary hospitals. Additionally, we did not include patients who received thoracic surgery within 30 days or patients with genetic predispositions (e.g., Duchenne-Muscular dystrophy, Birt-Hogg-Dube’s syndrome). Figure 1 shows the number of patients selected based on our inclusion and exclusion criteria. This study was approved by the Institutional Review Board of our institution (No.3-2021-0218). Patients’ consents were waived because this study was not related to treatments and patients’ clinical outcomes due to its retrospective design.

### 2.2. Study Definitions and Outcome Assessment

We reviewed the demographic variables (age, sex, Charlson Comorbidity Index, smoking history, body-mass index) of patients and pneumothorax characteristics, including location and history of previous pneumothorax through electronic medical records. The amount of pneumothorax was measured by using the Collins’s method via X-rays. Two thoracic surgeons calculated the amount, and the mean value was used to minimize any observer biases. Massive pneumothorax was defined as a case that occupied more than 50% of the ipsilateral thoracic cavity where pneumothorax occurred. When patients had a pneumothorax on both sides simultaneously, both sides were combined for the calculation. Additionally, chest computed tomography was reviewed during the hospitalization and evaluated whether re-expansion pulmonary edema occurred.

### 2.3. Treatment Protocols

Patients with minimal pneumothorax (less than 10%) who visited the emergency department or outpatient clinic usually received high-oxygen therapy. Oxygen was given by nasal cannula at 6 L/min, reservoir mask at 15 L/min, or humidified high-flow nasal cannula, and serial chest X-rays were performed after four hours. If pneumothorax increased compared to the initial event, chest tube insertion was considered using a 12Fr trocar (Y&B medics, Seoul, Korea). Chest tube insertion was generally recommended for patients with moderate to severe pneumothorax. If there was persistent air leakage or the lung did not fully expand, even with the chest tube inserted, an operation was recommended. Also, if a large bulla was observed on a chest computed tomography, surgery was recommended. In a case with re-expansion pulmonary edema, we applied supplement oxygen with cautious monitoring of the patients’ status and evaluated further treatment in case of severe deterioration. All patients with recurrent pneumothorax were usually treated surgically.

### 2.4. Statistical Analysis

Continuous variables (body-mass index, Charlson Comorbidity Index, height, operation time, hospital duration period) were described as the median and interquartile ranges (IQR), except weight, which was expressed as the mean and standard deviation. Categorical variables were indicated as characteristics or percentages of patients based on the group. As we analyzed the continuous variables, the Mann–Whitney test or two-sample t-test was used after testing for normality by the Shapiro–Wilk method. The Chi-square test, or Fisher’s exact test was used to analyze categorical variables. Logistic regression was performed to process univariate and multivariable risk factors for massive pneumothorax. Factors with a *p*-value less than 0.1 were used for multivariable analysis. We used R package version 4.0.5 for statistical analysis. Additionally, *p*-values of less than 0.05 were defined as significant.

### 2.5. Patient and Public Statement

Patients or the public were not involved in the design, conduct, reporting, or dissemination plans of our research.

## 3. Results

### 3.1. Patient Characteristics

**Figure 2:**
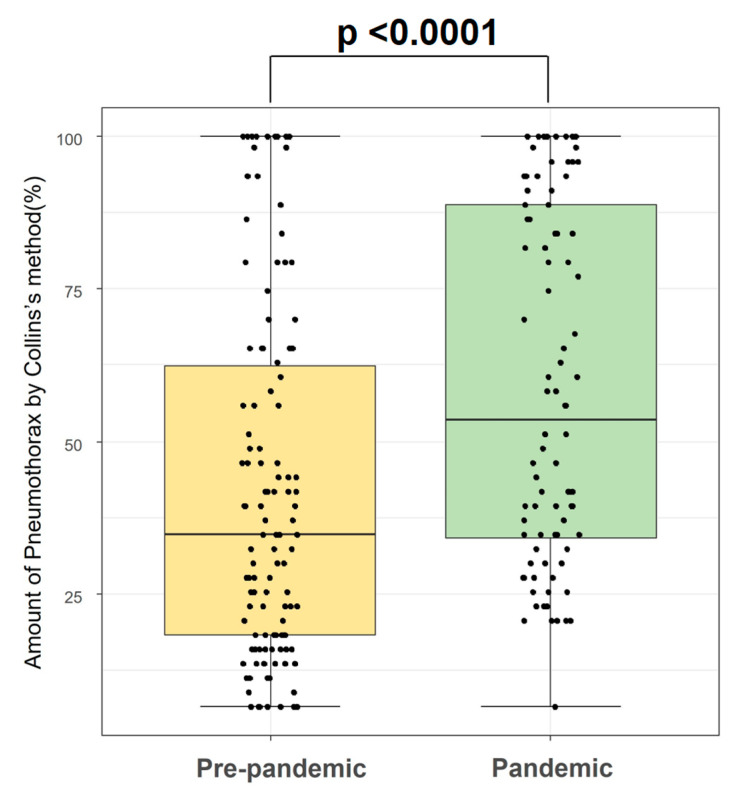
The amount of pneumothorax in pre-pandemic and pandemic periods.

A total of 210 patients were included in this study: 122 patients in the pre-pandemic group and 88 patients in the pandemic group. The median age [IQR] of the pre-pandemic group and pandemic group was 22.0 [18.0–36.75] and 20.0 [18.0–37.5] (*p* = 0.704), respectively. In both groups, most patients were male; 100 (82%) in the pre-pandemic group, and 75 (85.2%) in the pandemic group (*p* = 0.578). There was no significant difference in other demographic variables, including height, weight, and Charlson Comorbidity Index (Table 1). In terms of pneumothorax history, the location of the lesion was not statistically different, and the primary diagnosis was also comparable between the two groups; half of the cases were primary spontaneous pneumothorax (pre-pandemic: 59.8% vs. pandemic: 54.5%, *p* = 0.652). However, there was a significant difference in symptom onset to hospital arrival time (pre-pandemic: 1 day [IQR 0–2] vs. pandemic: 2 days [IQR 1–4], *p* = 0.00034). With respect to the amount of pneumothorax, it was significantly higher in the pandemic group (pre-pandemic: 34.75% [IQR 18.3–62.95] vs. pandemic: 53.55% [IQR 33.58–88.8], *p* < 0.0001) (Figure 2). In Figure 2, each patient’s pneumothorax amount was represented as a point in the jitter plot, and patients in the pandemic group were less distributed under 25% of the pneumothorax amount. Moreover, the pandemic group had more patients presenting with massive pneumothorax (the amount over 50%) (52.3% vs. 30.3%, *p* = 0.0016).

In treatment results, there was no major morbidity or mortality in both groups. However, the pandemic group had more re-expansion pulmonary edema (pre-pandemic: 11.5% vs. pandemic: 22.7%, *p* = 0.0366), and additional pleurodesis due to prolonged air leakage (pre-pandemic: 4.9% vs. pandemic: 15.9%, *p* = 0.0153) (Table 2).

### 3.2. Risk Factor Analysis for Massive Pneumothorax

The risk factors included demographic variables (age, sex, body-mass index, height, weight, smoking history), factors related to pneumothorax characteristics (location of the lesion, classification of diagnosis according to the British Thoracic Society guideline [14]), and the temporal aspect (whether it was before or during the pandemic). Multivariable analysis with logistic regression revealed that the pandemic period (OR: 2.70 [95% CI 1.49–4.90], *p* = 0.0011) and smoking history (OR: 3.23 [95% CI 1.68–6.22], *p* = 0.0004) were significant factors for massive pneumothorax (Table 3).

## 4. Discussion

Although there are several studies regarding the pneumothorax of COVID-19 patients [11,15,16,17], to the best of our knowledge, studies describing the impact of the pandemic on non-COVID-19-related pneumothorax patients are lacking. We tried to fill this gap and evaluated the impact of the pandemic on non-COVID-related pneumothorax.

When we evaluated the quantity of pneumothorax in each patient, it was found that patients came to the hospital with an increased amount of pneumothorax during the pandemic. As the severity of the shunt is dependent on the size of the pneumothorax, we could assert that patients presented with an aggravated status of pneumothorax since the pandemic started. If pneumothorax patients are presenting with a worsened condition due to a delayed hospital visit and care, they are exposed to a higher risk of major morbidity and mortality. This increased risk was observed as more re-expansion pulmonary edema among patients during the recovery process in this study, though severe life-threatening conditions did not occur. Pneumothorax patients showing up with worsened conditions may be attributable to the following three reasons.

Firstly, there was the hesitation to seek medical care or visit the hospital by many patients. Other studies have also reported a delayed presentation of stroke [8], myocardial infarction [18,19], appendicitis [6,20,21], and ectopic pregnancy [22]. Similarly, our study illustrates an exacerbated pneumothorax status as a consequence of delayed hospital visits due to the fear of the pandemic. As we stated, the difference in symptom-to-hospital arrival time between the two groups surely represents how patients are influenced by restrictive government policy during the pandemic. Though there were no death or complications during the pandemic, we need to assess the benefit and risk of these restrictive measures to prevent further collateral damage related to the pandemic.

Secondly, there was the effect of mask-wearing on airway resistance. South Korea is 1 of 28 countries with the highest compliance of mask-wearing; about 94% of South Koreans reported wearing face masks [23]. Several studies indicated that mask-wearing could induce increased airway resistance. Mapelli and their colleagues reported a significant worsening of cardiorespiratory parameters (forced expiratory volume in one second, forced vital capacity, oxygen uptake, carbon dioxide production) when wearing face masks at rest [24]. Lässing et al. also reported an increase in airway resistance when wearing surgical masks [25]. Taking these together, prolonged mask-wearing could result in a higher volume of air trapped in the thoracic cavity among pneumothorax patients.

Thirdly, there was an environmental aspect; factors such as temperature, atmospheric pressure, or the level of air pollution could impact the amount of pneumothorax. In 2018, Park et al. reported that high levels of air pollutants (ozone, nitric oxide, particulate matter (PM) ≤ 10 μm (PM10), and PM ≤ 2.5 μm (PM2.5)) were related to a high incidence of primary spontaneous pneumothorax in Seoul [26]. However, the impact of air pollution on pneumothorax during the pandemic is controversial; the nationwide study of air pollution in South Korea during the pandemic demonstrated lower levels of pollutants [27], but another study of the Seoul area showed an increased level of sulfur dioxide [28]. In several metropolitan areas, the lockdown did not relate to an improved level of air quality [29]. Furthermore, a recently published systematic review of the correlation between pneumothorax incidence and meteorologic factors showed that an increased temperature seems to be associated with pneumothorax incidence [30]. However, to the best of our knowledge, no study has shown the relationship between environmental factors and pneumothorax. As we currently do not have sufficient data about the temperature and the concentration of air pollutants around the area of our institution, a future study is warranted to evaluate the causal relationship between environmental factors and pneumothorax.

Considering all these findings, restrictive measures related to COVID-19 constituted a significant factor that caused patients to visit hospitals later than the pre-pandemic level. Additionally, there is insufficient evidence that the pandemic affected the pneumothorax’s pathophysiologic process related to pleural porosity or inflammation [31,32,33]. Although pneumothorax patients came to our hospital with a progressed status during the pandemic, we provided the highest quality of care. There was no mortality, and the overall hospital stay was not significantly different between the two periods. Therefore, as long as the health care providers are providing a certain level of quality of care, the treatment outcome may not be impacted by the pandemic, irrespective of the status of pneumothorax.

This study has several limitations. It is a retrospective study and was done in a single center located in one district of a metropolitan city. Therefore, it cannot be generalized to other regions and countries. In addition, this study cannot explain the causal relationship between the pneumothorax amount and restrictive government measures. Multicenter and multinational studies would be necessary to obtain a better understanding of the collateral impact of the pandemic.

## 5. Conclusions

The finding of this study suggest that general pneumothorax patients presented with an increased amount of pneumothorax during the pandemic, and more patients experienced re-expansion pulmonary edema in the course of treatments. The pandemic was also found to be related to the development of massive pneumothorax, but further studies investigating the underlying reasons for this may be warranted.

## Figures and Tables

**Table 1:** Baseline characteristics.

Variable	Pre-Pandemic *n* = 122 (%)	Pandemic *n* = 88 (%)	*p*-Value
Gender, No. (%)			0.578
Male	100 (82.0)	75 (85.2)	
Female	22 (18.0)	13 (14.8)	
Age, yr, median(IQR)	22 (18–36.75)	20 (18–37.5)	0.704
Charlson Comorbidity index, median (IQR)	0 (0–0)	0 (0–0)	0.818
Height, cm, median(IQR)	174 (169–178)	174 (169–180)	0.585
Weight, kg, mean (SD)	59.05 (8.74)	59.73 (9.67)	0.602
BMI, kg/m^2^, median(IQR)	20 (18–22)	20 (18–22)	0.692
Smoking history, No. (%)			1.00
Yes	33 (27.0)	23 (26.7)	
No	89 (73.0)	65 (73.3)	
Location of PNx, No. (%)			0.701
Right	64 (52.5)	42 (47.7)	
Left	57 (46.7)	46 (52.3)	
Both	1 (0.8)	0 (0.0)	
Diagnosis, No. (%)			0.652
Primary Spontanenous PNx	73 (59.8)	48 (54.5)	
Recurrent PNx	40 (32.8)	31 (35.2)	
Secondary PNx	9 (7.4)	9 (10.2)	
Onset to hospital time, day, median(IQR)	1 (0–2)	2 (1–4)	0.00034 **
PNx Amount, %, median(IQR)	34.75 (18.30–62.95)	53.55 (33.58–88.80)	<0.0001 **
Massive PNx, No. (%)	37 (30.3)	46 (52.3)	0.0016 *

*, *p* < 0.05; **, *p* < 0.001. BMI: Body-mass index, IQR: Interquartile range, SD: Standard deviation, PNx: Pneumothorax.

**Table 2:** Treatment outcomes in pre-pandemic and pandemic periods.

Variable	Pre-Pandemic *n* = 122	Pandemic *n* = 88	*p*-Value
Treatment, No, (%)			
Oxygen	0 (0.0)	3 (3.4)	0.0962
Chest Tube	38 (31.1)	30 (34.1)	
Operation	84 (68.9)	55 (62.5)	
Operation time, minutes, median(IQR)	34 (25–41.0)	33 (27–43.5)	0.552
Presence of Adhesion, No, (%)			0.182
Yes	20 (23.8)	19 (34.5)	
No	64 (76.2)	36 (65.5)	
Coverage of stapling site			0.186
None	2 (2.4)	1 (1.8)	
Single	23 (27.4)	23 (41.8)	
Double	59 (70.2)	31 (56.4)	
Surgical glue application			0.648
Yes	82 (97.6)	53 (96.4)	
No	2 (2.4)	2 (3.6)	
Intraoperative Pleurodesis	1 (1.2)	2 (3.6)	0.562
Hospital stay, days, median(IQR)	2 (2–3)	2 (2–2)	0.277
Re-expansion pulmonary edema, No. (%)	14 (11.5)	20 (22.7)	0.0366 *
Additional pleurodesis, No. (%)	6 (4.9)	14 (15.9)	0.0153 *

*, *p* < 0.05; IQR: Interquartile range.

**Table 3:** Univariate and multivariable risk factor analysis for massive pneumothorax.

Variables	Univariate Analysis		Multivariable Analysis	
	OR (95% CI)	*p*-Value	OR (95% CI)	*p*-Value
Pandemic era	2.52 (1.42–4.45)	0.0015 *	2.70 (1.49–4.90)	0.0011 *
Age	1.00 (0.99–1.02)	0.577		
BMI	1.07 (0.96–1.19)	0.222		
Recurrent PNx	0.54 (0.29–1.00)	0.0496 *	0.52 (0.27–1.01)	0.0524
Secondary PNx	0.60 (0.21–1.70)	0.337		
Height	0.99 (0.96–1.03)	0.594		
Female	0.89 (0.42–1.88)	0.752		
Leftside PNx	0.73 (0.42–1.28)	0.270		
Smoking History	3.02 (1.60–5.67)	0.0006 **	3.23 (1.68–6.22)	0.0004 **
Weight	1.01 (0.98–1.04)	0.545		

*, *p* < 0.05; **, *p* < 0.001; BMI: Body-mass index; PNx: Pneumothorax.

## Data Availability

The datasets used and/or analysed during the current study are available from the corresponding author on reasonable request.

## Abbreviations

BMI: Body-mass index; CI: confidence interval; IQR: interquartile range; OR: odds ratio; PM: particulate matter; PNx: pneumothorax; SD: standard deviation.
